# 3D Photogrammetry-Driven Craniofacial Analysis in Orthodontics: A Scoping Review of Recent Applications

**DOI:** 10.3390/bioengineering12111263

**Published:** 2025-11-18

**Authors:** Pui Ki Hung, Junqi Liu, Zhiyi Shan

**Affiliations:** Division of Paediatric Dentistry and Orthodontics, Faculty of Dentistry, The University of Hong Kong, Hong Kong SAR, China

**Keywords:** 3D photogrammetry, craniofacial analysis, orthodontics, facial scanning, anthropometry

## Abstract

(1) Background: The increasing utilization of three-dimensional (3D) photogrammetry has elevated craniofacial analysis to new dimensions. This scoping review seeks to provide a comprehensive overview of the current applications of 3D photogrammetry-supported craniofacial analysis within orthodontic practice, assess its technical superiority, and explore potential areas for enhancement. (2) Methods: A comprehensive search of the literature was carried across three electronic databases (PubMed, Web of Science, Embase). Two independent reviewers screened the articles and extracted data in accordance with the PRISMA-ScR guideline. The primary findings from the included articles were synthesized and analyzed qualitatively. (3) Results: A total of 479 records were obtained initially, with 53 articles ultimately included after removing duplicates and applying eligibility criteria. The application of 3D photogrammetry in craniofacial analysis has become prevalent in orthodontic practice, encompassing normative facial anthropometry, orthodontic problem finding, orthodontic treatment optimization, and treatment outcome evaluation. (4) Conclusion: 3D photogrammetry offers orthodontists a precise and efficient imaging technique for craniofacial analysis.

## 1. Introduction

Advancements in digital dentistry is irrevocably transforming modern dental practice and establishing itself as the new paradigm in dental healthcare. At the heart of the digital dentistry lies comprehensive digital imaging, which is essential in driving unprecedented precision and workflow optimization. Dental imaging is continuously undergoing transformative evolution, for example, progressing from conventional two-dimensional (2D) imaging to three-dimensional (3D) imaging. Compared with 2D imaging, the dimensional leap of 3D imaging can provide more spatial information and numerical precision in anatomical evaluation [1,2]. Among these advancements, 3D photogrammetry—a technique that reconstructs 3D surface models from overlapping 2D photographs using geometric algorithms—has emerged as a transformative tool. Unlike traditional methods, such as 2D photographs or 3D radiographs, 3D photogrammetry provides a comprehensive and radiation-free approach to capture high-fidelity craniofacial morphology. Its ability to quantify soft-tissue contours with high accuracy enables it to be a critical adjunct to conventional dental practice [3].

Orthodontic treatment aims to achieve dentofacial harmony through moving teeth [4]. Therefore, comprehensive craniofacial analysis, encompassing both hard tissue and soft tissue is essential for a high-quality orthodontic practice. The advent of cone-beam computed tomography (CBCT) has revolutionized hard tissue evaluation in orthodontic practice, improving treatment certainties. For example, observing the spatial relationship between tooth roots and cortical bone to assess the risk of bone dehiscence and fenestration during tooth movement, and evaluating the maturity of the midpalatal suture to provide guidance for skeletal maxillary expansion [5]. Although for soft tissue, the effect of CBCT is not satisfactory as it does not indicate true color and real skin texture [6]. In contrast 3D photogrammetry, which does not cause radiation exposure, is less time consuming, and provides data in three dimensions with color and texture is expected to act as a new robust modality for soft tissue analysis.

With the advent of 3D facial scanning technology, 3D photogrammetry-driven craniofacial analysis is not a novel concept and has been widely applied across many fields, particularly in craniomaxillofacial surgery [7]. However, its utilization in orthodontic practice remains less explored, primarily due to suboptimal imaging accuracy and the operational inefficiencies of the instruments. In recent years, driven by technological advancements, 3D photogrammetry-based craniofacial analysis is becoming more precise and accessible [8,9,10], therefore expanding its clinical applications in orthodontics. For instance, 3D photogrammetry systems have become increasingly accessible due to innovations in consumer-grade cameras [11,12], smartphone-based applications [8], and software [9]. The reduction in hardware costs compared to early commercial systems like 3dMD, enabled chairside imaging without specialized infrastructure, making the technology viable for rural or resource-limited practices. In addition, integration with other advanced technologies, such as artificial intelligence and multimodal imaging data integrations, has further redefined its role in modern orthodontic workflows.

The field of 3D facial photogrammetry has undergone a profound transformation, driven by innovations in camera systems, automated processing algorithms, and sophisticated analytic software. Consequently, there has been a surge in studies exploring its clinical utility in orthodontic diagnosis and treatment planning. However, this accelerated progress has outpaced the available scholarly synthesis. Existing review articles do not focus on the novel applications and technological capabilities specific to craniofacial analysis. It is necessary to map the current landscape, consolidate recent findings, and identify future directions for this dynamic tool. Therefore, this scoping review aims to overview the current applications of 3D photogrammetry-driven craniofacial analysis in orthodontics, discuss its advantages and limitations, and explore potential improvements, thereby giving new insights into future research and clinical applications.

## 2. Materials and Methods

### 2.1. Protocol

This scoping review was performed following the Preferred Reporting Items for Systematic Reviews and Meta-Analyses extension for Scoping Reviews (PRISMA-ScR) guidelines [13]. This study’s research question was: what are the current applications of 3D photogrammetry-driven craniofacial analysis in Orthodontics? A scoping search of MEDLINE (via PubMed) was first conducted to refine the search strategy. The protocol of this scoping review was registered on Open Science Forum (osf.io/gmc47) (accessed on 22 December 2024).

### 2.2. Eligibility Criteria

The inclusion criteria were constructed according to the PICOS format: P (Population): Human subjects including patients, healthcare providers, and lay individuals. I (Intervention): 3D photogrammetry-supported craniofacial analysis in clinical orthodontic practice, including combined orthodontic and orthognathic surgery. C (Comparison): Any imaging techniques utilized for craniofacial analysis, manual caliper measurement, or blank control. O (Outcomes): All types of outcomes associated with 3D photogrammetry-assisted craniofacial analysis. S (Study design): Original clinical studies published in English since January 2019, including but not limited to case reports, cross-sectional studies, etc.

Exclusion criteria were as follows: (1) Study not directly related to orthodontics (e.g., sleep apnea, alcohol syndrome, cleft). (2) Non-clinical studies, letters, expert opinions, and review papers. (3) Studies written in a language other than English. (4) Articles without full-text available.

### 2.3. Information Sources and Search

A formal comprehensive search of the literature was subsequently performed across three electronic databases (MEDLINE/PubMed, Web of Science and Embase) in December 2024. The second round of the search was conducted in June 2025 to collect updated papers. Given the absence of appropriate subject terms, keywords and their synonyms were specifically selected and combined for the literature search (Table S1).

### 2.4. Study Selection

Reference management and selection were conducted using the EndNote software (EndNote 21, Clarivate Philadelphia, PA, USA). After eliminating duplicate studies, two reviewers (HPK and LJQ) independently screened the titles, abstracts, full texts, and reference list of the included studies. Any disagreement was addressed through discussion or consultation with the third reviewer (SZY) until final consensus was achieved.

### 2.5. Data Extraction and Synthesis

The data extraction process was conducted by two reviewers (HPK and LJQ) independently. The collected information was organized into tables, including the author names, year of publication, aim of study, study population or sample size, type of 3D scan machine and software employed, and broad-based orthodontic outcome domain.

A comprehensive analysis of the extracted data was performed. The qualitative data synthesis was carried out in a narrative manner, detailing various applications of 3D photogrammetry-supported craniofacial analysis in orthodontics.

## 3. Results

### 3.1. Selections and Characteristics of Included Studies

**Figure 1:**
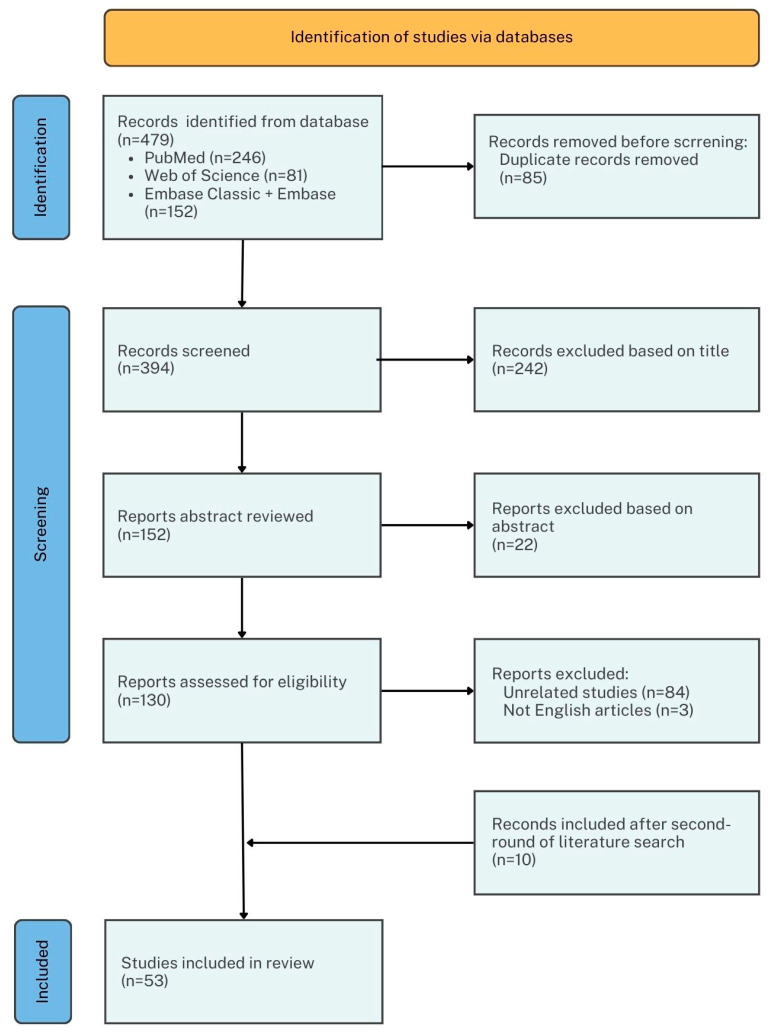
Flow chart of the workflow of identifying eligible studies.

The initial search strategy resulted in 479 records, 85 of which were excluded as duplicates. After removing duplicates, the titles and abstracts of 394 records were screened, leading to the exclusion of 264 records. Of the remaining 130 records undergoing full-text screening, 86 records were excluded for not fulfilling the eligibility criteria. Finally, 43 were selected for the qualitative analysis [14,15,16,17,18,19,20,21,22,23,24,25,26,27,28,29,30,31,32,33,34,35,36,37,38,39,40,41,42,43,44,45,46,47,48,49,50,51,52,53,54,55,56] (Figure 1). A second round of searching identified 77 additional records. After removing 20 duplicates, the titles and abstracts of 57 records were screened. A total of 42 records were excluded based on title and abstract and 5 were excluded after a full-text screening. A total of 10 were selected for the qualitative analysis at last [57,58,59,60,61,62,63,64,65,66]. (Tables S1 and S2)

The geographical distribution of the 53 included studies highlights a polycentric yet concentrated field of research. The work is led by a triad of major area contributors: China [21,23,24,26,28,33,36,38,43,47,48,50,57,58], Italy [18,30,37,44,51,55,66] and Taiwan [39,40,52,53,62,63], which together account for over half of all publications. Strong regional activity is also evident in East Asia (South Korea [27,54,56], Japan [20]) and Southern Europe (Greece [45], Spain [19], Türkiye [25,31,32,41,42]), reflecting a broad, global engagement with 3D facial imaging technology. This distribution underscores that the drive for innovation in this domain is not confined to a single region but is a shared priority across international academic and clinical communities, facilitating diverse perspectives and methodologies.

**Figure 2:**
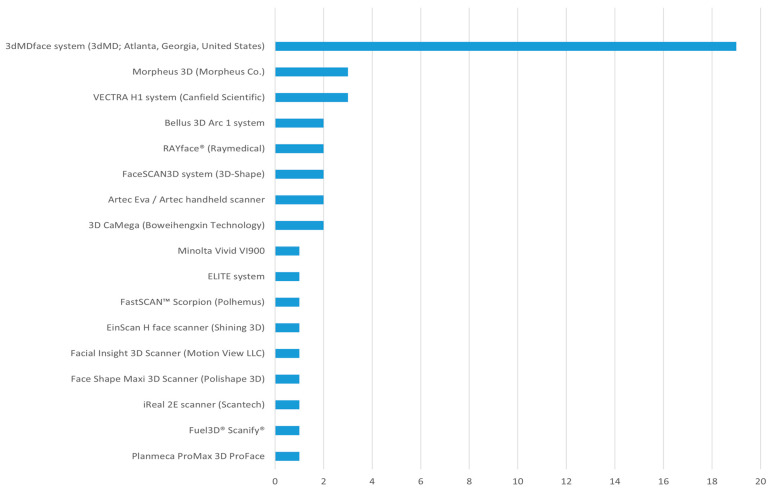
Bar chart of number of studies using different imaging tools.

The analysis of the 53 included studies revealed a clear predominance of specific technologies for data acquisition and processing. The 3dMDface system (3dMD LLC) was the overwhelmingly dominant imaging tool, employed in 19 studies (35.8%) [14,15,20,21,22,26,28,29,31,32,35,36,38,39,40,41,42,45,46,52,53,55,59,60,62,63,65] and was used to capture 1652 subjects, solidifying its status as the industry standard for high-resolution 3D facial stereophotogrammetry. Other imaging systems were also utilized, including the VECTRA H1 (Canfield Scientific) [16,17,51], Morpheus 3D [33,54,56], Artec Eva handheld scanner (Artec Eva^TM^; Artec Group, Luxembourg) [47,49], FaceSCAN3D (3D-Shape) [24,58] and Bellus 3D Arc 1 [23,57] (Figure 2).

**Figure 3:**
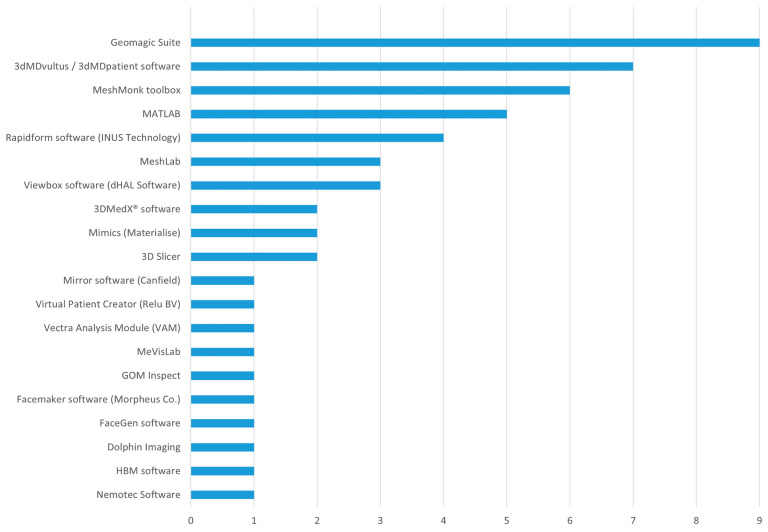
Bar chart showing number of studies using different softwares.

Software usage was more heterogeneous, reflecting a range of analytical approaches. The MeshMonk toolbox, frequently operated within the MATLAB environment, was used to process the largest cohort of subjects (*n* = 1091) [16,21,33,48,57,58,59,60,62], highlighting its central role in dense surface correspondence and geometric morphometric analysis. The proprietary Geomagic suite (e.g., Studio 2013, Essentials 2, Wrap 2015, Control, Qualify 12, Studio 2015) was the most frequently cited software family, used in nine studies for tasks like surface registration and deviation analysis [24,25,26,28,36,50,57,61,66]. The vendor-specific 3dMDvultus and 3dMDpatient software were also heavily employed for initial data processing and clinical assessment [31,32,41,42,46,55,59,62,63,65]. Other widely used software included Rapidform (INUS Technology) [14,15,43], Viewbox (dHAL Software) [22,29], 3D Slicer [44,64], and 3DMedX^®^ software [52,60]. A variety of specialized software packages were applied for specific tasks, such as Nemotec for anthropometric analysis [19], HBM [20] for constructing normative faces, GOM Inspect for deviation analysis [49]. This landscape demonstrates a methodological ecosystem reliant on a few dominant, high-fidelity imaging systems, paired with a diverse array of software ranging from commercial clinical packages to powerful open-source toolboxes for advanced computational research (Figure 3).

The 53 eligible publications can be categorized into the following four aspects: normative facial anthropometry [14,15,16,17,18,19,20], orthodontic problem finding [21,22,23,24,25,26,27,28,29,57,58], orthodontic treatment optimization [30,31,32,33,34,35,36,37,38,59,60], and treatment outcome evaluation [27,31,32,39,40,41,42,43,44,45,46,47,48,49,50,51,52,53,54,55,56,61,62,63,64,65,66].

### 3.2. Normative Facial Anthropometry

Eight publications were included regarding the use of 3D photogrammetry in normative anthropometry (Table 1). In these studies, 3D photogrammetry was used to analyze population-[14,15,16,19], age-[17,18] or sex-[19] specific facial features. When analyzing facial morphology, three of the studies generated average faces by aligning the facial scans using commercial softwares. From the generated average face, linear measurements, color histograms, surface areas, and shapes can be measured or calculated for normative facial anthropometry. Three studies identified landmarks manually or automatically by software to calculate a mean value with standard deviation for linear and angular measurements [17,18,19]. All studies calculate quantitative statistics except Tanikawa, C. et al. [20] who proposed the superimposition of average face and individual face scans for diagnostic purpose.

### 3.3. Orthodontic Problem Finding

Eleven studies have demonstrated the clinical utility of 3D photogrammetry for identifying various orthodontic problems, either as a standalone technique or in combination with conventional anthropometric methods (Table 2). Half of them focused on evaluating facial asymmetry [21,22,23,24,57,67] employing different approaches such as using spatially dense geometric morphometrics [21], overlapping 3D facial images along the midsagittal plane [22], constructing a 3D facial mask with an average facial model [23], constructing a 3D symmetry reference plane with a mathematical algorithm [24], and constructing a wireframe template with facial landmarks to calculate angle asymmetry [57]. Two studies discussed the analysis of the social smile, focusing on the relationship between smile characteristics and the surrounding hard and soft tissues [25,26]. One study explored the relationship between maxillary central incisors and the forehead [27], when another one evaluated the hard-soft relationship of the lower half of the face [28]. Mao et al. used machine learning to classify sagittal and vertical skeletal discrepancies in 3D facial scans [58]. The one remaining study investigated landmark identification for initial orthodontic diagnosis using a moving 3dMD face camera system, which captured 3D facial scans with recorded motions, enabling the analysis of dynamic facial expressions [29].

### 3.4. Orthodontic Treatment Optimization

Ten studies were included regarding treatment optimization (Table 3). Three-dimensional photogrammetry can be used to facilitate the manufacturing of appliances, continuous monitoring of the treatment’s progress or favoring treatment planning and patient communication. One of them focused on the use of 3D facial scanning on the manufacturing of a fully digital protraction facemask [30] and another three were about the continuous monitoring of facial changes during or after orthodontic treatment at fixed intervals [31,32,33]. One of the studies focused on the creation of virtual patients using a facial scan, an intraoral scan with a low-dose computed tomography that is based on artificial intelligence, which was assessed for its realism and usefulness for treatment planning and communication [34]. In addition to virtual patient creation, 3D facial scanning is an effective communication tool for facial aesthetic evaluation. Ponnusamy A et al. demonstrated this by using a generated 3D average face to assess perceptions of attractiveness among both dental professionals and laypersons [35]. Based on the 3D average face, different facial component such as face length, pogonion point, cheek volume or protraction of upper face can be modified to demonstrate the expected treatment outcome for better patient communication or facial aesthetic evaluation [36,37,38,59]. Berends, B. et al. developed a deep learning-based method to predict postoperative facial soft tissue outcomes following orthognathic surgery, which can not only improve treatment outcome, but also enhance communication between patients and orthodontists [60].

### 3.5. Treatment Outcome Evaluation

3D photogrammetry was commonly used to evaluate soft tissue changes after orthodontic treatments, both objectively and subjectively (Table 4). A total of twenty-seven studies were included regarding post-operative evaluation for orthodontic treatment [27,31,32,39,40,41,42,43,44,45,46,47,48,49,50,51,52,53,54,55,56,61,62,63,64,65,66]. Four studies reported subjective evaluation on post-operative facial attractiveness [39,40,62,63]. Other than subjective evaluation, the remaining included studies showed an objective outcome evaluation based on 3D photogrammetry, adopting different methods [27,31,32,41,42,43,44,45,46,47,48,49,50,51,52,53,54,55,56,61,64,65,66]. Twelve of them marked landmarks on the facial scan and directly measured and compared pre-operative and post-operative linear, angular, volumetric or ratio measurements [31,32,41,42,43,44,45,46,47,64,65,66]. Four of them marked specific landmarks and aligned pre- and post-operative face scans using a best-fit approach for 3D space deviation analysis to quantify volumetric changes [48,49,50,66]. Eight of them perform superimposition of two scans with or without a color-coded map for positive or negative change for post-operative evaluation [27,51,52,53,54,55,56,61].

## 4. Discussion

Three-dimensional photogrammetry has emerged as a transformative technology in orthodontics, enabling precise craniofacial analysis across multiple clinical domains. As indicated by this scoping review summarizing the latest five years of the literature, one of the most common applications of 3D photogrammetry lies in normative facial anthropometry, where the soft tissue measurement is elevated to 3D level, providing a more comprehensive and accurate assessment compared to traditional 2D measurements. It facilitates the creation of population-specific reference datasets for facial soft tissues, allowing clinicians to quantify deviations in symmetry, proportions, and growth patterns with high accuracy and zero radiation exposure [17,18,19]. Similarly, 3D photogrammetry excels in diagnosis by providing detailed 3D quantification of facial morphology (e.g., skeletal discrepancies, lip incompetence, asymmetry) through landmark identification and spatial analysis of scans. Moreover, it is often integrated with CBCT for comprehensive hard/soft tissue evaluation [22,28,67]. Treatment outcome evaluation was another well-validated use case, leveraging superimposition and color-mapped deviation analysis to objectively measure surgical or orthodontic outcomes (e.g., changes in lip volume, mandibular position, or asymmetry). This makes the evaluation of the treatment outcome more intuitive, thereby enhancing the communication between healthcare providers and patients [41,52,54]. Three-dimensional photogrammetry also plays a role in treatment optimization. While studies demonstrate promise in simulating aesthetic outcomes (e.g., virtual modifications of chin asymmetry, lip volume) and integrating scans for digital treatment planning [34,36,37], these applications face limitations in standardization and clinical integration. Overall, 3D photogrammetry significantly enhances the accuracy and efficiency of orthodontic assessment in established domains like anthropometry, diagnosis, and outcome tracking, etc., though its full potential in aesthetic-driven planning and workflow optimization requires further validation and technological refinement.

Benefit from 3D photogrammetry, the creation of a digital “virtual patient” is a transformative concept, moving beyond static records to a dynamic model central to modern care. This model begins with the establishment of normative databases. By aligning numerous scans using software like Rapidform and MeshLab, studies generate population-specific average faces that provide a quantitative baseline for diagnosing asymmetry and ethnic variations [14,15,16,20]. Beyond diagnosis, the virtual patient profoundly enhances treatment communication and aesthetic evaluation. The ability to digitally modify facial components—such as adjusting chin position or cheek volume on a 3D scan—allows patients to visualize potential outcomes, transforming subjective discussions into evidence-based conversations [35,36,38]. The most advanced application involves fully integrated treatment optimization, where facial scans were merged with intraoral and CBCT data to create a comprehensive digital twin. This enables the simulation of hard tissue movements, and their soft tissue impacts before treatment begins, which can be further advanced by deep learning algorithms that can predict postoperative outcomes [34,60].

Complementing 3D photogrammetry’s clinical utility is the pivotal role of specialized software and algorithms. The value of this technology is realized through tools that automate and enhance analysis. Software toolkits like MeshMonk enable automated landmarking and spatially dense geometric morphometrics, facilitating the detection of subtle asymmetries and complex morphological changes over time without manual subjectivity [21,33]. This capability extends to diagnosis, where machine learning models can classify skeletal discrepancies directly from 3D facial scans [58]. Furthermore, bespoke algorithms are being designed for specific orthodontic tasks. These include constructing mathematically derived symmetry reference planes for objective asymmetry assessment and performing best-fit superimposition of sequential scans to generate color-coded deviation maps [24,52,55]. These maps provide an intuitive, quantitative visual assessment of treatment progress, objectively measuring the efficacy of interventions.

Despite its capabilities, there are several limitations and areas for development in 3D photogrammetry that warrant consideration. A primary challenge lies in the lack of standardization and methodological heterogeneity. As evidenced by the wide array of software tools and techniques used across the reviewed studies (e.g., MeshMonk, Geomagic, Rapidform), there is no consensus on a universal protocol for landmark identification, mesh alignment, or asymmetry analysis. This variability may undermine the reproducibility of measurements and complicates the comparison of results across different studies and clinical settings. Reconstruction algorithms should also be improved to further enhance geometric fidelity, especially in capturing complex contours like the alar base or labiomental fold. Similarly, automated landmarking and classification systems for pathologies or facial types need to be trained on larger, more diverse datasets to improve their accuracy and clinical utility. The review also identifies a notable gap in the validation of advanced applications, particularly the “virtual patient” concept and dynamic (4D) capture. While studies like Jindanil et al. [34] demonstrate the technical feasibility of integrating multimodal scans, the clinical accuracy, workflow efficiency, and predictive power of these digital twins for comprehensive treatment simulation remain under-studied. Crucially, the reliability of soft-tissue predictions based on underlying skeletal movements, even with advanced AI as attempted by Berends et al. [60], is not yet fully established and demands extensive longitudinal validation. Similarly, the potential of 4D photogrammetry to assess functional movements is almost entirely unexplored, with only a single study by Coppola et al. [29] focusing on technical accuracy rather than clinical application for mastication or speech analysis.

This scoping review has several inherent methodological limitations that should be considered when interpreting the findings. First, consistent with the objective of mapping the breadth of evidence, we did not perform a formal quality appraisal or risk-of-bias assessment of the included studies. While this review successfully catalogs the diverse applications of 3D photogrammetry, it cannot make definitive conclusions regarding the efficacy, reliability, or accuracy of the reported techniques. The findings represent the scope of research activity rather than a synthesis of validated clinical outcomes. The literature search was conducted in three major databases (PubMed, Web of Science, Embase) to capture the core peer-reviewed literature. It is possible that relevant studies indexed in other databases (e.g., Scopus, Google Scholar) were missed, introducing a potential for selection bias. Finally, the narrative synthesis approach, while appropriate for the research question, precludes statistical pooling of data. Therefore, this review provides a comprehensive overview of the field’s landscape but does not offer depth on specific clinical outcomes, a task that would require a subsequent systematic review with meta-analysis.

## 5. Conclusions

Three-dimensional photogrammetry represents a paradigm shift in orthodontic practice, safety, and versatility in normative anthropometry, orthodontic diagnosis, simulating and evaluating treatment outcomes. As this technology evolves, its integration into orthodontic workflows helps to advance both functional precision and aesthetic harmony, ensuring treatments align with cultural ideals, and patient expectations. Future research should focus on standardizing landmark protocols, expanding population-specific databases, and integrating artificial intelligence to further automate and refine predictive simulations, solidifying 3D photogrammetry as a cornerstone of modern orthodontics.

## Figures and Tables

**Table 1:** Summary of the findings regarding the use of 3D photogrammetry in normative facial anthropometry.

Authors	Year of Publication	Sample Size	Aim	Imaging Tools (IT) and Software(S) Used	Parameters Measured
Bhaskar, E. and C. H. Kau [14]	2020	301	Determination of the differences in three-dimensional (3D) facial features in a population from Zimbabwe and the United States.	IT: 3dMDfaceTM system (3dMD; Atlanta, GA, USA) S: Rapidform software 2006, INUS Technology, Seoul, Republic of Korea (RF6)	Average faces of two genders were formed by aligning the facial scans. Linear measurements, color histograms, surface areas, and shapes.
Kau, C. H. et al. [15]	2019	371	Determination of gender dimorphism and facial morphological changes from adolescence to adulthood in African American and Caucasian populations.	IT: 3dMDface system (3dMD; Atlanta, GA, USA)/a laser-scanning system consisting of two high-resolution Minolta Vivid VI900 3D cameras (Konica Minolta, Osaka, Japan) S: Rapidform software 2006, INUS Technology, Seoul, Republic of Korea (RF6)	Images were combined to produce a male and female average face where linear measurements, color histograms, surface areas, and shapes were calculated.
Rajbhoj, A. A. et al. [16]	2022	129	Deriving descriptive statistics of 3D facial shape, lip and cheek muscle pressure in subjects of European descent with normal dental occlusion.	IT: VECTRA H1 imaging system (Canfield Scientific, Fairfield, NJ, USA) S; MeshLab (CNR-ISTI, Pisa, Italy), MeVisLab (MeVis Medical Solutions, Bremen, Germany)	Average faces derived from male and female children, adolescent and adults with orthodontically untreated normal occlusion were generated.
Mengoa, M. G. R. et al. [17]	2024	84	Evaluation and comparison of facial metrics in women aged 20–65 years using a 3D stereophotogrammetry system to establish standardized values for facial metric variations in different age subgroups.	IT: Vectra H1 3D stereophotogrammetry equipment (Canfield Scientific, Inc., Fairfield, CT, USA) S; Vectra Analysis Module Software (VAM elaboration, Canfield Scientific Inc. Fairfield, CT, USA)	Twenty-one landmarks were identified. Linear and angular measurements were obtained to calculate a mean value with standard deviation.
Cenzato, N. et al. [18]	2024	269	Analyzing the three-dimensional morphology of the faces of growing patients with Class I and II occlusions, focusing on children aged between 6 and 9 years old.	IT&S: ELITE system	Sixteen landmarks were identified to calculate angular and linear measurement, and distance ratios.
Menéndez López-Mateos, M.L. et al. [19]	2019	100	Analysis of faces of a sample of healthy European adults from southern Spain with normal occlusion in order to establish reference facial soft tissue anthropometric parameters in this specific geographic-ethnic population, as well as to analyze sexual dimorphism.	IT: Panmeca ProMax 3D ProFace (Planmeca USA, Inc.; Roselle, IL, USA) S: Nemotec Arnetts FAB Software, version 10.0 (Software Nemotec SL, Madrid, Spain)	Thirty landmarks described by FARKAS were identified to measure the height and width of face, nose, occlusal area, orolabial area and chin. Angular measurements and ratio measurements were made based on the identified landmarks. Mean value and standard deviation of each measurement were found.
Tanikawa, C. et al. [20]	2019	200	Quantifying and visualizing the 3D configuration of the soft tissues of the face at rest to facilitate a quantitative and instantaneous understanding of a patient’s static facial characteristics.	IT: 3dMDface system (3dMD; Atlanta, GA, USA) S: HBM software (National Institute of Advanced Industrial Science and Technology, Japan)	A wire mesh fitting was conducted based on the assignment of landmarks to each 3D facial image. Three-dimensional normative faces and the standard deviations for different measurements were found based on the mesh. Developed a system to compare the 3D normative face with patient’s own scan.

**Table 2:** Summary of the findings regarding the use of 3D photogrammetry in orthodontic problem finding.

Author	Year of Publication	Sample Size	Aim	Imaging Tools (IT) and Software (S) Used	Method
Fan, Y. et al. [21]	2022	86	Facial asymmetry assessment in skeletal Class III patients with spatially dense geometric morphometrics.	IT: 3dMD imaging system (3dMD Inc., Atlanta, GA, USA) S: Meshmonk toolbox	The pointwise surface-to-surface distance between original and mirror face was measured and visualized for the whole face after robust Procrustes superimposition. The degree of overall asymmetry in an individual was scored using a root-mean-squared-error.
Gkantidis, N. et al. [22]	2023	20	Facial asymmetry and midsagittal plane definition in 3D: A bias-free, automated method.	IT: 3dMD stereophotogrammetric camera (3dMDface system, 3dMD Inc., Atlanta, GA, USA) S: Viewbox 4 software (Version 4.1.0.1 BETA 64, dHAL Software, Kifissia, Greece)	midsagittal plane of the face was automatically defined at the midpoints of the contralateral corresponding vertices of the superimposed models and color coded distance maps were constructed.
Lyu, L. et al. [23]	2024	/	Design an objective method for identifying asymmetry issues.	IT: Bellus 3D Arc 1 system (Bellus3D, Inc., Campbell, CA, USA)	Used 3D facial images and landmark-based anthropometric analysis to construct an average model to create 3D facial mask to evaluate asymmetry.
Zhu, Y. J. et al. [24]	2022	30	Investigating and evaluating a novel mathematical algorithm based on power function weighted Procrustes analysis to determine 3D facial SRPs for patients with mandibular deviation.	IT: Face Scan 3D sensor system (3D-Shape Corp., Erlangen, Germany) S: Geomagic Studio 2013 (3D Systems Inc., Rock Hill, SC, USA)	Statistics and measurement analyses were used to comprehensively evaluate the clinical suitability of the power function weighted Procrustes analysis to construct the 3D symmetry reference plane.
Yang, G. et al. [57]	2025	24	Evaluating the accuracy and efficacy of a new wireframe template methodology in analyzing three-dimensional facial soft tissue asymmetry.	IT: Bellus 3D Arc 1 system (Bellus3D, Inc., Campbell, CA, USA) S: Geomagic Studio 2015 (Geomagic, Inc., Morrisville, NC, USA)/MeshLab (MeshLab 2020.12, 3D)/Procrustes Analysis (PA) algorithm software MATLAB R2019b (The MathWorks, Inc., Natick, MA, USA), Open-Source Nonrigid (OSN) software MATLAB R2019b, MATLAB R2019b, Open-Source Rigid Software (OSS) MATLAB R2019b, and OSN alignment program MeshMonk	Wireframe template was established by identifying 34 facial landmarks and then forming a template on the face, and the angle asymmetry index was automatically scored using the template.
Dindaroğlu, F. et al. [25]	2023	20	Evaluating social smile asymmetry in patients with unilateral impacted maxillary canine on 3D stereophotogrammetric images.	IT: Fuel3D^®^ Scanify^®^ S: Geomagic Essentials 2 reverse engineering software	Tissues around the smile area were divided into cheek, upper lip lateral and medial, and lower lip lateral and medial. Deviation margins and the total percentages of meshes deviations were calculated.
Li, H. et al. [26]	2019	50	Exploring the internal relationship between posed smile characteristics, lip position, and skeletal patterns in young women.	IT: 3dMDface system (3dMD; Atlanta, GA, USA) S: Geomagic Control software (3D Systems, Research Triangle Park, NC, USA)	Each subject was scanned with a posed smile. Interlabial gap, intercommissural width, and smile index were calculated. The changes in the lip landmarks in the vertical, sagittal, and coronal directions were investigated.
Cho, S. W. et al. [27]	2021	50	Evaluating and comparing the sagittal relationship between the maxillary central incisors and the forehead before and after orthodontic treatment.	IT: RAYface^®^ (Raymedical, Seongnam, Republic of Korea) S: Asahi Alphard 3030^®^ (Asahi Roentgen Ind., Co., Ltd., Kyoto, Japan).	Superimposition of the CBCT and 3D facial scan was carried out on the identical program to create a digital twin for facial analysis. Trichion/superion, glabella, maxillary incisor are located.
Wang, T. et al. [28]	2024	52	Clarifying the hard-soft tissue relationships of the lower 1/3 of the face in skeletal Class II-hyperdivergent patients compared with those in Class I-normodivergent patients using network analysis.	IT: 3dMD imaging system (3dMD, Atlanta, GA, USA), NewTom Scanner (NewTom AG, Marburg, Germany) S: Geomagic Studio 11.0 software (Raindrop Geomagic, Inc., NC, USA)	Cone-beam computed tomography and three-dimensional facial scans were taken and superimposed, on which landmarks were identified manually, and their coordinate values were used for network analysis.
B. Mao et al. [58]	2025	435	Using machine learning (ML) to classify sagittal and vertical skeletal discrepancies in 3D facial scans, as well as to evaluate shape variability.	IT: 3D optical FaceSCAN3D system (3D-Shape, Erlangen, Germany) S: MATLAB (MATLAB R2018b; The MathWorks Inc., Natick, MA, USA), using the MeshMonk toolbox	Sixty-three facial landmarks were determined and geometric measurements formed by these points along with demographic characteristics were used in the machine learning models for sagittal and vertical classification.
Coppola, G. et al. [29]	2024	/	Compare various recording speeds for three standardized movements using the 3dMDface camera system, to assess its accuracy and reliability.	IT: 3dMD imaging system (3dMD, Atlanta, GA, USA), UR5e Universals Robots, Universal Robots GmbH, München, Germany S: Viewbox 4 Software (version 4.1.0.1 BETA 64, dHAL software, Kifisia, Greece)	A linear and two circular movements were performed using a 3D-printed cube mounted on a robotic arm, accuracy and reliability were evaluated.

**Table 3:** Summary of the findings regarding the use of 3D photogrammetry in orthodontic treatment optimization.

Author	Year of Publication	Sample Size	Aim	Imagining Tools (IT) and Software(S) Used	Method
Fiorillo, G. et al. [30]	2024	/	Describing the fully digital workflow for the production design and manufacturing of a fully digital protraction facemask.	/	Shell mask is virtually drawn and finished to guarantee 22 support zones on the forehead, cheekbones, and chin. The mask is prototyped by stereolithography using biocompatible resins.
Akan, B. and İ. Veli [31]	2020	20	Evaluating the effects of the Forsus fatigue-resistant device EZ2 appliance (3M Unitek, Monrovia, Calif) on facial soft tissues.	IT: 3dMD Face (3dMD Ltd., Atlanta, GA, USA) S: 3dMD Vultus software (3dMD, Atlanta, GA, USA)/SAS 9.3 Software (SAS Institute, Cary, NC, USA)	Three-dimensional facial scanning images were obtained with 3dMD Face. Cephalometric radiographic images were taken before placement of the appliance (T0), immediately after removal (T1), and at the 6-month (T2) follow-up after the removal of the appliance.
Yüksel Coşkun, E. and E. Esenlik [32]	2020	32	Comparing adolescent and post-adolescent growth periods regarding the effectiveness of conventional activator appliance in patients.	IT: 3dMDface system (3dMDface LLC, Atlanta, GA, USA) S: 3dMDface Vultus^®^ software (3dMDface Vultus^®^ software version 2.3.0.2, 3dMDface, Atlanta, GA, USA)	Projections of the lips and the chin and volumetric measurements of the lip and the mandibular area were assessed using three-dimensional photogrammetry.
Hou, S. Y. et al. [33]	2021	43	Investigating changes in facial morphology during the first six months of orthodontic treatment among adult females receiving orthodontic treatment.	IT: Morpheus 3D, Republic of Korea S: IDAV Landmark Editor v.3.0.0.6 /MeshMonk toolbox of MATLAB (R2018b)	Spatially dense facial landmarks were digitized. An anthropometric mask was mapped to each facial image resulting in 3D facial quasi-landmarks. Three-dimensional geometric morphometrics and multivariate statistics were used to investigate changes.
Jindanil, T. et al. [34]	2024	20	Constructing a virtual patient (VP) using facial scan, intraoral scan, and low-dose computed tomography scab based on an Artificial intelligence.	IT: iReal 2E structured light-based scanner (Scantech, Hangzhou, China)/Emerald S device (Planmeca, Helsinki, Finland)/SOMATOM Force unit (SIEMENS, Munich, Germany) S: Virtual Patient Creator (version 1.0.0 March 2022, Relu BV, Leuven, Belgium)	The accuracy of the virtual patients created using AI-driven, AI-refined and semiautomated registration was analyzed. User satisfaction was assessed through a survey on the virtual patient’s realism and usefulness for treatment planning and communication.
Ponnusamy, A. et al. [35]	2020	375	Establishment of the most pleasing computer-generated 3D facial form using a community-based sample population.	IT: 3dMD camera (3dMD Inc., GA, USA)	Seven average faces for each gender were generated, arranged from most concave to most convex profile and rated by dentists, oral surgeons, plastic surgeons, orthodontists and laypeople for attractiveness to find out most pleasant face.
Dong, T. et al. [36]	2020	221	Assessing the influence of chin asymmetry on perceived facial aesthetics, investigating the cognitive boundaries of chin asymmetry among orthodontists, general dentists, and laypersons, and providing quantitative reference for clinical treatment.	IT: 3dMDface system (3dMD, Atlanta, GA, USA) S: Geomagic Wrap 2015 (3D Systems)	Soft tissue pogonion point of the facial image were altered in 2 mm increments from 0 to 12 mm to produce 14 images and were rated by 66 orthodontists, 89 general dentists, and 66 laypersons to determine whether treatment was needed.
Fastuca, R. et al. [37]	2022	105	Investigating the influence of different facial components on the perception of facial attractiveness of patients in orthodontists, laypersons and patients with 2D and 3D.	S: FaceGen software (Singular Inversions Inc., Toronto, ON, Canada)/Dolphin Imaging & Management Solutions, Chatsworth, CA, USA	Four facial components were modified (face length, lip volume, nose size and cheekbone contour) and the resulting simulations were used to obtain 2D figures and 3D videos, which were evaluated in a survey by orthodontists, patients, and laypersons.
Feng, J. et al. [38]	2019	30	Investigating the influence of cheek volume on facial esthetics judged by orthodontists and non-specialists.	IT: 3dMDface system (3dMD, Atlanta, GA, USA)	A 25-year-old female’s natural and smiling face was captured by 3D stereophotogrammetry. Cheek volume of the 3D image was altered to different degrees three-dimensionally.
Nguyen, K. T. et al. [59]	2025	34	Investigating the upper lip changes induced by a simulated protraction of upper front teeth.	IT: 3dMDtrio, 3dMDLLC, Atlanta, GA, USA S: 3dMDpa-tient; 3dMD/Meshlab (Meshlab v1.3.4,ISTI—CNR, Pisa, Italy)	Simulated protraction of upper front teeth was achieved and stereophotogrammetry was used to assess lip changes in three dimensions.
Berends, B. et al. [60]	2025	458	Developing and validating a deep learning-based method to predict postoperative facial soft tissue outcomes in real time for various orthognathic procedures.	IT: 3 dMD’s 5-pod 3dMDhead systems (3dMDCranial, 3 dMD, Atlanta, GA, USA) International Inc, Hatfield, PA, USA) S: MeshMonk algorithm and DiffusionNets, 3DMedX^®^ software (version: 1.2.40.0, 3D Lab Radboudumc, Nijmegen, The Netherlands)	A deep learning-based method was developed based on 3D photographs for the real-time prediction of the effects of maxilla, mandible, and chin displacements on facial soft tissue following orthognathic surgery.

**Table 4:** Summary of the findings regarding the use of 3D photogrammetry in post-operative outcome evaluation.

Author	Year of Publications	Sample Size	Aim of Study	Imaging Tools (IT) and Software(S) Used	Method of Evaluation
Akan, B. and İ. Veli [31]	2020	20	Evaluating the effects of the Forsus fatigue-resistant device EZ2 appliance (3M Unitek, Monrovia, Calif) on facial soft tissues.	IT: 3dMD Face (3dMD Ltd., Atlanta, GA, USA) S: 3dMD Vultus software (3dMD, Atlanta, GA, USA)/SAS 9.3 Software (SAS Institute, Cary, NC, USA)	Three-dimensional facial scanning images were obtained with 3dMD Face. Cephalometric radiographic images were taken before placement of the appliance (T0), immediately after removal (T1), and at the 6-month (T2) follow-up after the removal of the appliance.
Yüksel Coşkun, E. and E. Esenlik [32]	2020	32	Comparing adolescent and post-adolescent growth periods regarding the effectiveness of conventional activator appliance in patients.	IT: 3dMDface system (3dMDface LLC, Atlanta, GA, USA) S: 3dMDface Vultus^®^ software (3dMDface Vultus^®^ software version 2.3.0.2, 3dMDface, Atlanta, GA, USA)	Projections of the lips and the chin and volumetric measurements of the lip and the mandibular area were assessed using three-dimensional photogrammetry.
Güler, Ö. and S. Malkoç [41]	2020	60	Evaluating the effects of three different fixed or removable functional appliances on the soft tissue changes in patients with Class II Division 1 malocclusion using 3-dimensional images.	IT&S: 3dMD Face system (3dMd, Atlanta, GA, USA)	Lower facial width, volumetric change in mandible, total facial height, convexity angle, and facial depth.
Akan, B. et al. [42]	2021	32	Evaluating the effects of tooth-borne and tooth-bone-borne rapid maxillary expansion on soft tissue with stereophotogrammetry.	IT: 3dMD Face imaging system (3dMD, Atlanta, GA, USA) S: 3dMDvultus analysis program (3dMD, Atlanta, GA, USA)	Changes in soft tissues before RME (T0) and post-retention (T1) period were evaluated by stereophotogrammetry. Linear and angular measurements were performed.
Liu, Z. Y. et al. [43]	2019	47	Investigating the three-dimensional lip vermilion changes after extraction and non-extraction orthodontic treatment in female adult patients and explore the correlation between lip vermilion changes and incisor changes.	IT: 3D CaMega; Boweihengxin Technology Inc., Beijing, China S: Rapidform 2009 software (Inus Technology)	Nine angles, seven distances, and the surface area of the lip vermilion were measured by using pre- and post-treatment three-dimensional facial scans. Linear and angular measurements of incisors were performed on lateral cephalograms.
Marino Merlo, M. et al. [44]	2024	28	Comparing the effects on facial soft tissues produced by maxillary expansion generated by rapid maxillary expansion versus slow maxillary expansion.	IT: Face Shape Maxi 3D Scanner (Polishape 3D srl, Bari, Italy) S: 3D Slicer (open-source)	Difference in the facial tissue changes in the nasal area measured on facial 3D images captured immediately before application of the expander (T0) and after one year of retention, immediately after the expander removal (T1). (Nasal columella width, mouth width, nasal tip angle, upper lip angle, and lower lip angle).
Sitaropoulou, V. et al. [45]	2020	36	Evaluating the skeletal, dental, and soft tissue effects of the alternating rapid maxillary expansions and constrictions (Alt-RAMEC) protocol combined with a facemask in prepubertal patients.	IT: 3dMDface system (3dMD LLC, Atlanta, GA, USA) S: MIMICs version 17.0 (Materialize Interactive Medical Image Control Systems, Leuven, Belgium)	Soft tissue landmarks identified and compared on T0 and T1 3D scans.
Nike, E. et al. [46]	2023	101	Evaluation of facial soft tissue asymmetric changes in Class III patients after orthognathic surgery using three-dimensional stereophotogrammetry.	IT: 3dMDtrio stereophotogrammetry system (3dMD LLC, Atlanta, GA, USA) S: 3dMDvultus version 2.5.0.1 (3dMD LLC/3dMD Patient version 4.1 (3dMD LLC)	Three-dimensional photographs were acquired using the 3dMDtrio stereophotogrammetry system, and 21 anthropometric landmark positions were evaluated at three time points: before surgery (T0), 6 months (T1) and 12 months (T2) after surgery.
Shi, Y. et al. [47]	2022	30	Evaluating facial soft and hard tissue changes, individually and relative to each other, in patients with skeletal class III deformity after bimaxillary surgery using 3D photos obtained by white light scanning.	IT: Artec Eva hand-held 3D scanner S: CMF PROPLAN 3.0 (Materialise)/Artec studio 12.0 software	Three-dimensional color-coded map was analyzed to assess both skeletal and soft tissue changes between T0 and T1. Changes in the 3D coordinates of each anatomical landmark were analyzed using Student’s *t*-test.
Molla, N. et al. [64]	2025	32	Evaluate the effects of maxillary expansion over a period of 12 months on facial soft tissue measurements in children aged 7–11 years.	IT: Facial Insight 3D Scanner (Motion View LLC, Chattanooga, TN, USA) S: 3D Slicer software (version 4.11.20210226, Boston, MA, USA)/OrthoInsight 3D software (Version 7.7.5570; Motion View LLC, Chattanooga, TN, USA)	Soft tissue analysis after rapid maxillary expansion using landmarks and linear and angular measurements on 3D photographs.
Nike, E. et al. [65]	2025	54	Examine changes in facial soft tissue asymmetry over time in patients after Class II orthognathic surgery using 3D stereophotogrammetry.	IT: 3dMD Trio stereophotogrammetry system (3dMD LLC, Atlanta, GA, USA) S: 3dMD Vultus version 2.5.0.1 (3dMD LLC)/edited using 3dMD Patient version 4.1 software (3dMD LLC)	Evaluation of 21 anthropometric landmark positions was conducted before surgery (T0), 6 months (T1), and 12 months (T2) after surgery.
Pellitteri, F. et al. [66]	2025	45	Comparing the soft tissue changes in pretreatment and posttreatment facial scans of patients who had undergone various orthopedic treatments vs. a control group of untreated growing patients.	IT: EinScan H face scanner (70565; Shining 3D Technology GmbH, Stuttgart, Germany) S: Geometric Control X software (3D Systems, Rock Hill, SC)	After best-fit scan alignment, a 3-dimensional analysis of soft tissue changes was performed, comparing 3D reference points and 8 areas on T0 and T1 scans.
Mao, B. et al. [48]	2023	19	Investigating temporomandibular joint stability and 3D facial changes in class II hyperdivergent patients with stable idiopathic condylar resorption after orthodontic camouflage treatment.	IT: 3D optical FaceSCAN3D system (3D-Shape) S: MATLAB (MATLAB R2018b, MathWorks) with the MeshMonk toolbox	3D facial heatmaps were used to illustrate facial changes, and the 3D deviations of landmarks were calculated.
Alkhayer, A. et al. [49]	2021	25	Assessing soft tissue changes in the face after six months of retention following Rapid Maxillary Expansion.	IT: structured-light 3D handheld scanner (Artec EvaTM; Artec Group, Luxembourg) S: Artec Eva V.12/GOM Inspect Evaluation Software, Capture 3D, Inc., Santa Ana, CA, USA	Linear and angular measurements were performed, and 3D deviation analyses were performed for six morphological regions of the face.
Shen, L. H. et al. [50]	2021	42	Evaluating 3D morphological changes after orthodontic extraction treatment in lip vermilion of adult females with dentoalveolar protrusion using a structured light-based scanner.	IT: 3D CaMega; Boweihengxin Technology Inc., Beijing, China S: Geomagic Qualify 12 (3D Systems, Rock Hill, SC, USA)	Six landmarks, three linear measurements and three area measurements were measured. The spatial deviations of three volumetric measurements (upper, lower, and total vermilion) were constructed for quantitative analysis.
Cho, S. W. et al. [27]	2021	50	Evaluating and comparing the sagittal relationship between the maxillary central incisors and the forehead before and after orthodontic treatment.	IT: RAYface^®^ (Raymedical, Seongnam, Republic of Korea) S: Asahi Alphard 3030^®^ (Asahi Roentgen Ind., Co., Ltd., Kyoto, Japan).	Superimposition of the CBCT and 3D facial scan was carried out on the identical program to create a digital twin for facial analysis. Trichion/superion, glabella, maxillary incisor were located.
Da Pozzo, F. et al. [51]	2020	41	Compare the severity of facial asymmetry of class III malocclusion patient with Class I occlusion patient, and pre-op vs. post-op.	IT: VECTRA system S: Mirror software (Canfield Scientific)	Images of the hemi-faces of the subjects were divided into thirds (upper, middle, lower), mirrored and superimposed to their contralateral ones.
Chen, Y. H. et al. [52]	2024	37	Comparing facial 3D soft tissue changes in subjects with Class III deformities who underwent bimaxillary clockwise and counterclockwise rotational orthognathic surgery.	IT: 3dMD facial, Atlantic, USA S: 3DMedX^®^	Preoperative and 9-month follow-up CBCT and 3D stereophotogrammetry were obtained, superimposed, and quantified for skeletal movements and soft tissue changes in six facial regions.
Keardkhong, P. et al. [53]	2023	60	Comparing regional soft tissue changes between patients with class III overbite and open bite deformities treated with bimaxillary surgery involving clockwise and counterclockwise mandibular setback.	IT: 3dMDface System (3dMD Inc., Atlanta, GA, USA)	Combined cone-beam CT scans and 3D facial photographs preoperative and at least 1-year postoperative were taken to assess the soft tissue change (superimposed 3D images for direct comparison).
Kim, K. A. et al. [54]	2019	28	Three-dimensional soft tissue changes according to skeletal changes after mandibular setback surgery by using cone-beam computed tomography and a structured light scanner.	IT: Morpheus 3D Neo; Morpheus, Gyoung-gi, Republic of Korea S: Morpheus 3D software	To use an identical 3D coordinate system, superimposition was performed, and nine skeletal and 18 soft tissue landmarks were identified. Changes in the landmarks and correlation coefficients and ratios between hard and soft tissue changes were evaluated.
Rongo, R. et al. [55]	2021	46	Assessing the soft tissue changes in orthodontic extraction and non-extraction patients on 3D stereophotogrammetric images.	IT: white-light scanner 3dMD (3dMD) S: 3dMDVultusSoftware	After placing 19 landmarks, 15 measurements were obtained Superimposition of 3D images visualized with color-coded maps to show changes.
Choi, T. H. et al. [56]	2021	18	Evaluating the linear, angular, and volumetric changes in soft tissue after clockwise repositioning of the maxillo-mandibular complex in skeletal class III patients using 3D stereophotogrammetry and to determine the correlation between changes in the skeletal and soft tissue variables.	IT: Morpheus 3D; Morpheus Co, Gyeonggi, Republic of Korea S: Facemaker software (Facemaker; Morpheus Co)	Lateral cephalograms and 3D photographs taken before and 6 months after surgery were compared.
Simões, J. C. M. et al. [61]	2025	46	Analyzing changes in masticatory function and 3D facial soft tissue in patients with Class II and Class III DFD after orthognathic surgery.	IT: FastSCAN™ Scorpion laser scanning system (Polhemus, Inc., Colchester, CT, USA) S: FastSCAN™/Geomagic Studio (Geomagic, Research Triangle Park, NC, USA)	Twelve patients who completed the treatment protocol were evaluated 12 months post-surgery (T2). The patient groups were compared with each other and with a control group (CG) of 25 healthy subjects.
Denadai, R. et al. [39]	2020	70	Evaluating the impacts of orthognathic surgery on the patient’s facial appearance and apparent age, from surgeon and laymen perspective.	IT: 3dMDface system (3dMD; Atlanta, GA, USA)	Seven blinded plastic surgeons rated all photographs for apparent facial aesthetic and age scales. The FACE-Q data from 57 matched normal individuals were adopted for comparative analyses.
Denadai, R. et al. [40]	2019	84	Evaluating orthognathic surgery outcomes using pre- and post-OGS patients’ FACE-Q reports, and a three-dimensional facial photograph-based panel assessment of facial appearance.	IT: 3dMDface system (3dMD; Atlanta, GA, USA)	Ninety-six blinded layperson and orthodontic and surgical professional raters and verified whether there were correlations between these outcome measurement tools.
Chiang, W.C. et al. [62]	2025	135	Assessing facial smile attractiveness before and after orthognathic surgery using a transfer learning model.	IT: 3dMDhand™ System Product Specifications (3dMD Inc, Atlanta, GA, USA) S: 3dMD Vultus (3dMD LLC)/3-matic (CAD Software, Materialise, Belgium)/MATLABsoftware (MathWorks, Natick, MA, USA)	A total of 38 evaluators, comprising plastic surgeons, orthodontists, and research assistants, were instructed to rate the facial smile attractiveness of 3D facial images using a 5-point Likert scale.
Hu, I. et al. [63]	2025	86	Investigating the hard tissue and soft tissue characteristics related to favorable facial attractiveness outcomes of Class II Twin-block treatment to select suitable patients for this therapy.	IT: 3dMD face, Atlanta, GA, USA S: 3dMD vultus program	Post-Twin-block treatment facial attractiveness was assessed by seven orthodontists using visual analog scale scores based on 3D photographs.

## Data Availability

The original contributions presented in the study are included in the article, further inquiries can be directed to the corresponding author.

## Supplementary Materials

The following supporting information can be downloaded at: https://www.mdpi.com/article/10.3390/bioengineering12111263/s1, Table S1: Search strategies of each database and number of results for first round search; Table S2: Search strategies of each database and number of results for second round search.

## Abbreviations

The following abbreviations are used in this manuscript:

2D	Two-dimensional
3D	Three-dimensional
CBCT	Cone beam computed tomography
PRISMA-ScR	Systematic Reviews and Meta-Analyses extension for Scoping Reviews
IT	Imaging tools
S	Software

## References

[1] Gao P.C., Zhao Z.Q., Chen Y.L., Zhao Y., Xie L.Z., Yan B., Wang L. (2019). Accuracy of three-dimensional camera system based on stereophotography on photographic acquisition of deformity facial images. Hua Xi Kou Qiang Yi Xue Za Zhi.

[2] Aljawad H., Lim H.J., Lee K.C. (2023). Anthropometric Comparison of 3-Dimensional Facial Scan Taken with a Low-Cost Facial Scanner with Cone-Beam Computed Tomography Scan. J. Craniofac. Surg..

[3] Gašparović B., Morelato L., Lenac K., Mauša G., Zhurov A., Katić V. (2023). Comparing Direct Measurements and Three-Dimensional (3D) Scans for Evaluating Facial Soft Tissue. Sensors.

[4] Spalj S., Slaj M., Varga S., Strujic M., Slaj M. (2010). Perception of orthodontic treatment need in children and adolescents. Eur. J. Orthod..

[5] Angelieri F., Cevidanes L.H., Franchi L., Gonçalves J.R., Benavides E., McNamara J.A. (2013). Midpalatal suture maturation: Classification method for individual assessment before rapid maxillary expansion. Am. J. Orthod. Dentofac. Orthop..

[6] Kau C.H., Richmond S., Incrapera A., English J., Xia J.J. (2007). Three-dimensional surface acquisition systems for the study of facial morphology and their application to maxillofacial surgery. Int. J. Med. Robot..

[7] Doan J., Finlay N., Lee A., Ethell T., Gandedkar N., Darendeliler M.A. (2023). The accuracy (trueness and precision) of Bellus3DARC-7 and an in-vivo analysis of intra and inter-examiner reliability of digital and manual anthropometry. Aust. Orthod. J..

[8] Andrews J., Alwafi A., Bichu Y.M., Pliska B.T., Mostafa N., Zou B. (2023). Validation of three-dimensional facial imaging captured with smartphone-based photogrammetry application in comparison to stereophotogrammetry system. Heliyon.

[9] Barone S., Antonelli A., Salviati M., Greco V., Bennardo F., Becker K., Giudice A., Simeone M. (2024). Accuracy Assessment of EM3D App-Based 3D Facial Scanning Compared to Cone Beam Computed Tomography. Dent. J..

[10] Pellitteri F., Scisciola F., Cremonini F., Baciliero M., Lombardo L. (2023). Accuracy of 3D facial scans: A comparison of three different scanning system in an in vivo study. Prog. Orthod..

[11] Mao B., Li J., Tian Y., Zhou Y. (2022). The accuracy of a three-dimensional face model reconstructing method based on conventional clinical two-dimensional photos. BMC Oral. Health.

[12] Pan F., Liu J., Cen Y., Chen Y., Cai R., Zhao Z., Liao W., Wang J. (2022). Accuracy of RGB-D camera-based and stereophotogrammetric facial scanners: A comparative study. J. Dent..

[13] Tricco A.C., Lillie E., Zarin W., O’Brien K.K., Colquhoun H., Levac D., Moher D., Peters M.D.J., Horsley T., Weeks L. (2018). PRISMA Extension for Scoping Reviews (PRISMA-ScR): Checklist and Explanation. Ann. Intern. Med..

[14] Bhaskar E., Kau C.H. (2020). A Comparison of 3D Facial Features in a Population from Zimbabwe and United States. Eur. J. Dent..

[15] Kau C.H., Wang J., Davis M. (2019). A Cross-Sectional Study to Understand 3D Facial Differences in a Population of African Americans and Caucasians. Eur. J. Dent..

[16] Rajbhoj A.A., Matthews H., Doucet K., Claes P., Willems G., Begnoni G., Cadenas de Llano-Pérula M. (2022). Age- and sex-related differences in 3D facial shape and muscle pressure in subjects with normal occlusion. Comput. Biol. Med..

[17] Mengoa M.G.R., Garcia A., Fioravanti K.S., Neppelenbroek K.H., Oliveira T.M., Sforza C., Soares S. (2024). Facial morphology analysis of Caucasian Brazilian adult women using stereophotogrammetry. Braz. Oral. Res..

[18] Cenzato N., Farronato M., Tartaglia F.C., Giannini L., Inchingolo A.M., Dipalma G., Maspero C., Inchingolo F. (2024). Soft Tissue Facial Morphology in Growing Patients with Different Occlusal Classes. J. Pers. Med..

[19] Menéndez López-Mateos M.L., Carreño-Carreño J., Palma J.C., Alarcón J.A., Menéndez López-Mateos C., Menéndez-Núñez M. (2019). Three-dimensional photographic analysis of the face in European adults from southern Spain with normal occlusion: Reference anthropometric measurements. BMC Oral. Health.

[20] Tanikawa C., Akcam M.O., Takada K. (2019). Quantifying faces three-dimensionally in orthodontic practice. J. Craniomaxillofac. Surg..

[21] Fan Y., He W., Chen G., Song G., Matthews H., Claes P., Jiang R., Xu T. (2022). Facial asymmetry assessment in skeletal Class III patients with spatially-dense geometric morphometrics. Eur. J. Orthod..

[22] Gkantidis N., Opacic J., Kanavakis G., Katsaros C., Halazonetis D. (2023). Facial asymmetry and midsagittal plane definition in 3D: A bias-free, automated method. PLoS ONE.

[23] Lyu L., Zhang M.J., Wen A.N., Wang S., Zhao Y.J., Yong W., Yu T.T., Liu D. (2024). 3D facial mask for facial asymmetry diagnosis. Heliyon.

[24] Zhu Y., Fu X., Zhang L., Zheng S., Wen A., Xiao N., Wang Y., Zhao Y. (2022). A mathematical algorithm of the facial symmetry plane: Application to mandibular deformity 3D facial data. J. Anat..

[25] Dindaroğlu F., Fırıncıoğulları E.C., Duran G.S. (2023). Three-dimensional evaluation of social smile asymmetry in patients with unilateral impacted maxillary canine: A 3D stereophotogrammetry study. Clin. Oral. Investig..

[26] Li H., Cao T., Zhou H., Hou Y. (2019). Lip position analysis of young women with different skeletal patterns during posed smiling using 3-dimensional stereophotogrammetry. Am. J. Orthod. Dentofac. Orthop..

[27] Cho S.W., Byun S.H., Yi S., Jang W.S., Kim J.C., Park I.Y., Yang B.E. (2021). Sagittal Relationship between the Maxillary Central Incisors and the Forehead in Digital Twins of Korean Adult Females. J. Pers. Med..

[28] Wang T., Nie K., Fan Y., Chen G., Xu K., Han B., Pei Y., Song G., Xu T. (2024). Network analysis of three-dimensional hard-soft tissue relationships in the lower 1/3 of the face: Skeletal Class I-normodivergent malocclusion versus Class II-hyperdivergent malocclusion. BMC Oral. Health.

[29] Coppola G., Hänggi D., Cassina G., Verna C., Gkantidis N., Kanavakis G. (2024). Three-dimensional video recordings: Accuracy, reliability, clinical and research guidelines—Reliability assessment of a 4D camera. Orthod. Craniofac. Res..

[30] Fiorillo G., Garrisi L., Mandelli A., Arnò F., Mandelli G., Gastaldi G. (2024). Design and manufacturing of a fully digital protraction facemask. AJO-DO Clin. Companion.

[31] Akan B., Veli İ. (2020). Evaluation of soft-tissue changes in young adults treated with the Forsus fatigue-resistant device. Am. J. Orthod. Dentofac. Orthop..

[32] Yüksel Coşkun E., Esenlik E. (2020). A Prospective Study Comparing Adolescent and Post-Adolescent Periods Regarding Effects of Activator Appliance in Patients with Class II Mandibular Retrognathia by Using 3dMDface Analysis and Cephalometry. Med. Sci. Monit..

[33] Hou S.Y., Zhou W., Dai H., Wong H.M., Wen Y.F., Zhou J. (2021). Soft tissue facial changes among adult females during alignment stage of orthodontic treatment: A 3D geometric morphometric study. BMC Oral. Health.

[34] Jindanil T., Burlacu-Vatamanu O.E., Meyns J., Meewis J., Fontenele R.C., Perula M.C.L., Jacobs R. (2024). Automated orofacial virtual patient creation: A proof of concept. J. Dent..

[35] Ponnusamy A., Goonewardene M.S., Mian A., Eastwood P., Rea A., Islam S. (2020). Facial soft tissue norms in Caucasians using an innovative three-dimensional approach. Australas. Orthod. J..

[36] Dong T., Ye N., Yuan L., Wu S., Xia L., Fang B. (2020). Assessing the Influence of Chin Asymmetry on Perceived Facial Esthetics with 3-Dimensional Images. J. Oral. Maxillofac. Surg..

[37] Fastuca R., Beccarini T., Rossi O., Zecca P.A., Caprioglio A. (2022). Influence of facial components in class III malocclusion esthetic perception of orthodontists, patients, and laypersons. J. Orofac. Orthop..

[38] Feng J., Yu H., Yin Y., Yan Y., Wang Z., Bai D., Han X. (2019). Esthetic evaluation of facial cheek volume: A study using 3D stereophotogrammetry. Angle Orthod..

[39] Denadai R., Chou P.Y., Su Y.Y., Lin H.H., Ho C.T., Lo L.J. (2020). The Impacts of Orthognathic Surgery on the Facial Appearance and Age Perception of Patients Presenting Skeletal Class III Deformity: An Outcome Study Using the FACE-Q Report and Surgical Professional-Based Panel Assessment. Plast. Reconstr. Surg..

[40] Denadai R., Chou P.Y., Su Y.Y., Lo C.C., Lin H.H., Ho C.T., Lo L.J. (2019). Facial Appearance and Psychosocial Features in Orthognathic Surgery: A FACE-Q- and 3D Facial Image-Based Comparative Study of Patient-, Clinician-, and Lay-Observer-Reported Outcomes. J. Clin. Med..

[41] Güler Ö., Malkoç S. (2020). Comparison of facial soft tissue changes after treatment with 3 different functional appliances. Am. J. Orthod. Dentofac. Orthop..

[42] Akan B., Gökçe G., Şahan A.O., Veli İ. (2021). Tooth-borne versus tooth-bone-borne rapid maxillary expanders according to a stereophotogrammetric evaluation of facial soft tissues: A randomized clinical trial. Orthod. Craniofac. Res..

[43] Liu Z.Y., Yu J., Dai F.F., Jiang R.P., Xu T.M. (2019). Three-dimensional changes in lip vermilion morphology of adult female patients after extraction and non-extraction orthodontic treatment. Korean J. Orthod..

[44] Marino Merlo M., Quiroga Souki B., Nieri M., Bonanno A., Giuntini V., McNamara J.A., Franchi L. (2024). Comparison of the effects on facial soft tissues produced by rapid and slow maxillary expansion using stereophotogrammetry: A randomized clinical trial. Prog. Orthod..

[45] Sitaropoulou V., Yilmaz H.N., Yilmaz B., Kucukkeles N. (2020). Three-dimensional evaluation of treatment results of the Alt-RAMEC and facemask protocol in growing patients. J. Orofac. Orthop..

[46] Nike E., Radzins O., Pirttiniemi P., Vuollo V., Slaidina A., Abeltins A. (2023). Evaluation of facial soft tissue asymmetric changes in Class III patients after orthognathic surgery using three-dimensional stereophotogrammetry. Int. J. Oral. Maxillofac. Surg..

[47] Shi Y., Liu S., Shao X., Zong C., Bai S., Yang Y., Liu Y., Shang H., Tian L. (2022). Facial changes in patients with skeletal class III deformity after bimaxillary surgery: An evaluation based on three-dimensional photographs registered with computed tomography. Br. J. Oral. Maxillofac. Surg..

[48] Mao B., Tian Y., Li J., Zhou Y., Wang X. (2023). A quantitative analysis of facial changes after orthodontic treatment with vertical control in patients with idiopathic condylar resorption. Orthod. Craniofac. Res..

[49] Alkhayer A., Becsei R., Hegedűs L., Párkányi L., Piffkó J., Braunitzer G., Segatto E. (2021). Evaluation of the Soft Tissue Changes after Rapid Maxillary Expansion Using a Handheld Three-Dimensional Scanner: A Prospective Study. Int. J. Environ. Res. Public Health.

[50] Shen L.H., Xie T.Y., Jiang R.P., Jiang Y.R., Chen G., Xu T.M., Han B. (2021). Measurement of three-dimensional changes in lip vermilion in adult female patients after orthodontic extraction: A retrospective longitudinal study. Head Face Med..

[51] Da Pozzo F., Gibelli D., Beltramini G.A., Dolci C., Giannì A.B., Sforza C. (2020). The Effect of Orthognathic Surgery on Soft-Tissue Facial Asymmetry: A Longitudinal Three-Dimensional Analysis. J. Craniofac. Surg..

[52] Chen Y.H., Baan F., Bruggink R., Ko E.W., Bergé S., Xi T. (2024). Clockwise versus counterclockwise rotation in bimaxillary surgery: 3D analysis of facial soft tissue outcomes. Oral. Maxillofac. Surg..

[53] Keardkhong P., Chen Y.F., Yao C.F., Chen Y.A., Liao Y.F., Chen Y.R. (2023). Comparison of regional soft tissue changes after bimaxillary rotational surgery between class III deformity with overbite and open bite: A 3D imaging analysis. Biomed. J..

[54] Kim K.A., Chang Y.J., Lee S.H., An H.J., Park K.H. (2019). Three-dimensional soft tissue changes according to skeletal changes after mandibular setback surgery by using cone-beam computed tomography and a structured light scanner. Prog. Orthod..

[55] Rongo R., Nissen L., Leroy C., Michelotti A., Cattaneo P.M., Cornelis M.A. (2021). Three-dimensional soft tissue changes in orthodontic extraction and non-extraction patients: A prospective study. Orthod. Craniofac. Res..

[56] Choi T.H., Kim S.H., Yun P.Y., Kim Y.K., Lee N.K. (2021). Soft Tissue Changes After Clockwise Rotation of Maxillo-Mandibular Complex in Class III Patients: Three-Dimensional Stereophotogrammetric Evaluation. J. Craniofac. Surg..

[57] Yang G., Lyu L., Wen A., Zhao Y., Wang Y., Li J., Yan H., Zhang M., Yu Y., Yu T. (2025). Comparison of Mirroring and Overlapping Analysis and Three-Dimensional Soft Tissue Spatial Angle Wireframe Template in Evaluating Facial Asymmetry. Bioengineering.

[58] Mao B., Tian Y., Xiao Y., Li J., Zhou Y., Wang X. (2025). Classification of skeletal discrepancies by machine learning based on three-dimensional facial scans. Int. J. Oral. Maxillofac. Surg..

[59] Nguyen K.T., Farella M., Bennani V., Mei L. (2025). Effect of Biomechanical Properties of Perioral Soft Tissues on Lip Response to Simulated Protraction of Upper Front Teeth. J. Oral. Rehabil..

[60] Berends B., Bielevelt F., Baan F., Schreurs R., Maal T., Xi T., de Jong G. (2025). Soft-tissue prediction based on 3D photographs for virtual surgery planning of orthognathic surgery. Comput. Biol. Med..

[61] Simões J.C.M., Garcia D.M., De Mello-Filho F.V., De Felício C.M., Trawitzki L.V.V. (2025). Masticatory function and three-dimensional facial morphology of soft tissues: One year after orthognathic surgery. Arch. Oral. Biol..

[62] Chiang W.C., Chen H.L., Lin H.H. (2025). Automated 3D facial smile attractiveness assessment before and after orthognathic surgery using transfer learning: A preliminary study. J. Plast. Reconstr. Aesthet. Surg..

[63] Hu J., Wang C., Li Q. (2025). Characteristics of skeletal Class II adolescents with favorable facial attractiveness outcomes based on 3D photos after Twin-block treatment. J. Stomatol. Oral. Maxillofac. Surg..

[64] Molla N., Oh H., Heo G., Catunda R., Lagravère M. (2025). Comparison of soft tissue facial changes in patients 7–11 years of age with and without maxillary expansion utilizing CBCTs and 3D facial scans: A preliminary study. Int. Orthod..

[65] Nike E., Radzins O., Vuollo V., Slaidina A., Abeltins A. (2025). Changes in Facial Soft Tissue Asymmetry in Class II Patients One Year After Orthognathic Surgery. J. Clin. Med..

[66] Pellitteri F., Albertini P., Brucculeri L., Cremonini F., Guiducci D., Falconi V., Lombardo L. (2025). Soft tissue changes during orthopedic therapy: An in vivo 3-dimensional facial scan study. Am. J. Orthod. Dentofac. Orthop..

[67] Kwon S.M., Hwang J.J., Jung Y.H., Cho B.H., Lee K.J., Hwang C.J., Choi S.H. (2019). Similarity index for intuitive assessment of three-dimensional facial asymmetry. Sci. Rep..

