# Supplementary material for: 3D Photogrammetry-Driven Craniofacial Analysis in Orthodontics: A Scoping Review of Recent Applications
Source: Bioengineering (Basel). 2025 Nov 18;12(11):1263. doi: 10.3390/bioengineering12111263 (PMC12650070; doi:10.3390/bioengineering12111263)
Supplement: Supplementary file 1 [file bioengineering-12-01263-s001.zip › bioengineering-3945822-Supplementary Materials.pdf]

Table S1. Search strategies of each database and number of results for first round search.

Database	Keywords	No. of Results
Pubmed	((“2019/01/01”[Date-Publication]: “2024/12/31”[Date-Publication])) AND ((((“Three-dimensional photo*”) OR (“Three-dimensional fac*”) OR (“3D photo*”) OR (“3D fac*”) OR (photogrammetry*) OR (“Facial scan*”)) AND ((“Orthodont*”))))	246
Web of Science	(((“Three-dimensional photo*”) OR (“Three-dimensional fac*”) OR (“3D photo*”) OR (“3D fac*”) OR (photogrammetry*) OR (“Facial scan*”)) AND ((“Orthodont*”)))	81
Embase	1 Three-dimensional photo*.mp. 690<br>2 Three-dimensional fac*.mp. 548<br>3 3D photo*.mp. 1058<br>4 3D fac*.mp. 1090<br>5 exp photogrammetry/ 1843<br>6 ortho*.mp. 713765<br>7 craniofacial.mp. 54006<br>8 1 or 2 or 3 or 4 or 5 4688<br>9 6 or 7 762454<br>10 8 and 9 845<br>11 limit 10 to yr = “2019–2024” 421<br>12 limit 11 to full text 152	152

Table S2. Search strategies of each database and number of results for second round search.

Database	Keywords	No. of Results
Pubmed	((“2025/01/01”[Date-Publication] : “2025/06/30”[Date-Publication])) AND ((((“Three-dimensional photo*”) OR (“Three-dimensional fac*”) OR (“3D photo*”) OR (“3D fac*”) OR (photogrammetry*) OR (“Facial scan*”)) AND ((“Orthodont*”))))	46
Web of Science	(((“Three-dimensional photo*”) OR (“Three-dimensional fac*”) OR (“3D photo*”) OR (“3D fac*”) OR (photogrammetry*) OR (“Facial scan*”)) AND ((“Orthodont*”)))	15
Embase	1 Three-dimensional photo*.mp. 690<br>2 Three-dimensional fac*.mp. 548<br>3 3D photo*.mp. 1058<br>4 3D fac*.mp. 1090<br>5 exp photogrammetry/ 1843<br>6 ortho*.mp. 713765<br>7 craniofacial.mp. 54006<br>8 1 or 2 or 3 or 4 or 5 4688<br>9 6 or 7 762454<br>10 8 and 9 845<br>11 limit 10 to yr = “2025” 421<br>12 limit 11 to full text 152	16
